# Demand structure prediction and adaptation path of rural teachers in western China under population change

**DOI:** 10.1371/journal.pone.0331907

**Published:** 2025-09-18

**Authors:** Yang Lv, Binyu Hao, Xuemei Zhang, Min Wu

**Affiliations:** 1 College of Teachers, Chengdu University, Chengdu, China; 2 School of Public Administration, Sichuan University, Chengdu, China; International University of Business Agriculture and Technology, BANGLADESH

## Abstract

The construction of a rural teaching workforce is a key factor in promoting educational equity and the development of high-quality education. We employ the Leslie model and the age-specific grade progression rate method to estimate a population forecasting model. Using data from the seventh national census and regional statistical yearbooks and accounting for educational centralization and population trends, we forecast rural preschool, primary, and junior high teacher demand in 12 western Chinese provinces (2024–2040) on the basis of school-age population changes. The results show that (1) population changes have led to a decline in the allocation of rural education resources in western China; (2) the decrease in the school-age population has led to a continuous contraction of the teacher team structure, with a more pronounced decline in the preschool education stage; for example, rural student numbers in Gansu are projected to decline by 35% between 2024 and 2040, contributing to a 28% drop in primary teacher demand under constant policy conditions; and (3) the decline in teacher demand at all educational stages has exacerbated the imbalance in the allocation of educational resources. To address shrinking school-age populations and rural teacher imbalances, we recommend adjusting staffing standards on the basis of demographic forecasts, strengthening cross-stage professional development with clear pathways and incentives, and modernizing training through blended mentoring and data-driven coaching to ensure sustainable education quality.

## Introduction

The distribution of teachers’ resources has an important influence on the quality of education, as it ensures equitable access to high-quality education and encourages socioeconomic growth across the globe. Teachers’ demand is inextricably connected to trends in demographics, including population growth, birth rates and patterns of migration. Globally, urbanization trends and changes in population composition have had substantial effects on education worldwide [1]. Declining birth rates have led to school consolidations and reduced teacher shortages across many developed nations; however, in developing regions, rapid population growth has created an acute teacher shortage [2].

Significant changes have occurred in China’s population structure. They were primarily the result of China’s birth control program, which was initiated in the late 1970s. The one-child policy (1979--2015) and the resulting demographic changes have resulted in higher birth rates but now fewer school-age children. However, the rapid growth of urbanization has resulted in the depopulation of rural areas, which has led to a rise in the gap in educational resource distribution between rural and urban regions [3]. This demographic shift has led to difficulties in rural teacher supply, particularly in the western part of China, where infrastructure and economic constraints make the problem more difficult [4]. The construction of a rural teaching workforce is an important foundation for achieving educational equity and high-quality development. China’s Education Modernization 2035 clearly proposes addressing the problem of teacher resource shortages and promoting the development of a teaching workforce toward professionalization and high quality [5]. As a key area for educational development in China, ensuring the rational allocation of teacher resources is particularly critical for the modernization of rural education in western China.

In the western part of China, the school-age population has been plummeting for approximately a decade, while provisions for teacher resources have been heavily oversupplied. Urban schools struggle with increasing student enrollments engendered by migration, whereas the opposite is true for many rural schools, resulting in poor teacher distribution [6]. For almost a decade, the number of school-aged children in the western region of China has been declining, even as teaching resources have been overabundant. Moreover, migration has led to population growth in urban areas, which has resulted in many city schools facing a rising tide of new students. Most rural schools, however, have found the opposite to be true: their numbers have been decreasing [7]. This situation is a direct result of uneven migration. It is also a consequence of policy issues related to where teachers are recruited and to whom they are sent. Displacement is bad enough without the additional salt in the wound that comes from the conventional wisdom about the reliability of models for predicting supply and demand over the next ten years. It is essential to accurately predict rural teacher demand to maintain education systems that can adapt not only to demographic changes but also to economic and social changes [8]. This is especially true in western China, where reduced population growth and the influx of people to urban areas have cut school enrollments in the hinterlands beyond the narrow band of towns that line major highways [9]. Consequently, even though a small number of recently graduated teachers have now entered the ranks of working educators, not enough teachers serve too many underresourced and poorly staffed classrooms. Forecasting these figures is critical not only for recruitment but also for ensuring that any teacher who steps into a classroom is prepared not only to live and work in a difficult-to-access area but also to confront the many challenges associated with their teaching careers [10]. With the continuous decline in the birth rate and the rapid advancement of urbanization, the size of the rural school-age population in western China has decreased significantly, and the disparity between the supply and demand of teacher resources has become increasingly evident, leading to delayed adjustments and widening urban‒rural gaps. The dynamic changes in the school-age population and the mismatch between the supply and demand of educational resources have made it difficult for rural schools to meet teaching needs both in terms of teacher numbers and workforce stability [11]. In this study, we fill a crucial gap in the literature by combining demographic forecasting models with educational planning frameworks to predict the demand of rural teachers in western China. These findings are helpful for policymakers, educators, and researchers interested in aligning teacher supply with trends in the K-12 education student population. This research contributes to more general debates about sustainable education systems, regional equity and the need for responsive policy mechanisms by tracing the effects of population decline on rural education institutions. Moreover, this study is important in contributing to the global knowledge of adaptation strategies to rural education.

Research on teacher resource availability in the face of demographic change has focused on different dimensions of the problem. For example, Haepp and Lyu [12] investigated the impact of declining birth rates on school closures and teacher reallocation via data from China. They reported that the greatest loss of students came among rural schools, which now have surplus teachers in some areas and shortages across others. Finally, Chen et al. [13] investigated the effects of urban migration on rural teacher retention and reported that socioeconomic factors make it difficult for many rural cites to attract and retain qualified teachers. The necessity of predictive models in educational planning has been one of the game changers internationally. In sub-Saharan Africa, where there are high levels of population growth and the FRA teachers needed, Bongaarts [14] used population projection methods to estimate the needed number of teachers needed over a recent period. In comparison, European studies have focused on optimizing decreasing teacher demand—mostly through teacher retirement incentives and multigrade teaching models [15]. However, the need for region-specific research that combines demographic forecasting and teacher demand analysis persists, especially in China’s western rural area. In this respect, this study attempts to fill this gap through a systematic approach to teacher demand projection at multiple education levels, using the Leslie model and grade progression ratio method.

Limited academic attention has been given to the issue of the demand for teachers in western China, although previous studies might have focused on national or provincial demand forecasting in China, therefore not addressing rural specific conditions. Some studies have investigated how the dwindling number of people affects educational outcomes [16–18], but none have attempted to integrate demographic projections with forecasts of teacher demand. The absence of this crucial analysis means that we have no clear picture of how the rural education problem is likely to evolve over the next decade—either in a favorable or an unfavorable direction. Moreover, several strategies have been employed, with a fair measure of success, to counteract the imbalanced rural teacher supply and demand. However, we have very little hard evidence that any of these approaches are working. This study addresses these gaps by using an integrated modeling approach to combine population forecasting and teacher demand. This study also provides a deeper understanding of how demographic shifts impact rural education and offers specific policy guidance for optimizing teacher allocations. Therefore, this study intends to address three research questions: (a) What are the projected trends in student populations in rural western China over the next few decades? (b) How do these demographic changes impact the demand for rural teachers in terms of quantity and specialization? (c) What adaptation strategies can be implemented to align teacher supply with the projected demand in these regions? Thus, this study develops a predictive model for the demand structure of rural teachers in western China, considering current and projected population change trends. Additionally, this study proposes adaptation pathways to ensure that teacher supply aligns with future educational needs, thereby enhancing the quality and equity of education in these regions.

This research is novel in its method and policy implications. This study applies advanced demographic modeling techniques that enable us to go beyond static teacher allocation analysis and provide more responsive modeling for teacher allocation. The results could help shape national and regional educational policies and ensure that planning for the teacher workforce keeps pace with changes in the population. This research is important for rural development and educational planning. First, it develops a comprehensive forecasting model that integrates demographic projections with a detailed analysis of teacher demand. This makes for a better and more flexible approach to using educational resources. Second, it analyzes the need for rural teachers at a much finer level of detail than heretofore, at the regional level, in western China, addressing a critical gap in the research. Finally, it proposes policy strategies that are not only sensible but also adaptable to other areas with similar demographic patterns and educational needs.

In the context of ongoing efforts to modernize education, scientifically forecasting future teacher demand and formulating regional resource allocation strategies are urgent tasks that need to be addressed. This study examines twelve provinces (municipalities and autonomous regions) in western China as the research focus. On the basis of data from the seventh national census and relevant statistical yearbooks of each region and incorporating population dynamics trends, the Leslie model and the grade progression ratio method are used to systematically predict the demand structure for rural preschools, primary schools, and junior high school teachers from 2024-- 2040. The goal is to provide a scientific basis for the rational allocation of rural education resources and to promote the high-quality development of rural education in western China. The findings provide evidence-based input for national teacher deployment strategies. By forecasting rural teacher demand across regions and education stages, the results can guide the Ministry of Education in adjusting teacher allocation norms, planning interprovincial deployments, and aligning teacher training quotas with projected needs—supporting a more balanced and efficient teacher distribution nationwide.

## Literature review

### Theoretical foundations of teacher resource allocation

The allocation of educational resources should be based on population forecasting. By reviewing the literature, it can be concluded that the theories and methods of population forecasting can be divided into two categories.

The first category consists of forecasting methods based on classical statistical theory and mathematical models. These methods typically assume that the overall population growth trend follows a specific mathematical formula [19–21], such as the Markov chain, regression analysis, or gray system theory. One advantage of these methods is their high stability, as well as their strict and rigorous mathematical derivation process.

The second category includes inference methods that draw upon demographic principles that leverage fundamental parameters such as birth, death, and migration rates for forecasting. These approaches include single-factor, multifactor, and cohort factor methods. They are especially appropriate for short-term and medium-term forecasts of population size and structure, taking as their foundation a large amount of historical data. At present, population prediction software such as the CPPS and PADIS-INT uses calculation rules mainly on the basis of the cohort factor method. For example, Wang et al. [22] used PADIS-INT to forecast trends in population growth and estimate the demand for different types of preschool teachers in Shanghai between 2024 and 2050.

The allocation of teacher resources is an essential factor in the planning process in the education system and is supported by multiple theoretical approaches. According to human capital theory, investing in education—especially in terms of teacher quality—increases productivity and therefore economic outcomes for both the individual and society [23]. This approach highlights strategic teacher placement as a means to maximize education gains.

The theory of supply and demand can also shed light on how teacher labor markets operate. Education provision is dependent upon the relationship between the educational requirements of the population and the availability of qualified teachers [24]. A serious imbalance could lead to shortages of teachers or a glut of them, both of which create unique problems for education systems. Resource allocation is paramount in equity and efficiency. Equity is concerned with the equitable allocation of resources and the access to quality education they provide for all students, and efficiency is related to the effectiveness of resource use in generating the highest educational outcomes [25]. Ensuring that these principles work in harmony is crucial for policymakers seeking to address disparities, particularly in communities characterized by a variety of socioeconomic contexts. Theories of population change (such as demographic transition models) explain how changes in birth rates, death rates, and patterns of migration affect the demand for education (and thus the need for teachers) [26]. Teacher workforce planning is particularly important in regions that have undergone notable demographic transformations; however, it necessitates a nuanced comprehension of current and future demographic trends.

### Teacher resource allocation from a population perspective

Given the rapid development of education and changing demographic trends, scientifically establishing standards for teacher resource allocation plays an important role in meeting the educational needs of the school-age population and promoting the high-quality development of the teaching workforce. A review of existing research shows that teacher resource allocation methods can be divided into three categories.

#### Policy-based approaches to teacher resource allocation.

The first category is based on policy-oriented standardized allocation. Most studies refer to the provisions on teacher staffing and student‒teacher ratios in relevant national or local policy documents and make appropriate adjustments on the basis of anticipated trends in educational development to predict teacher demand. For example, Qin and Zong [8] predicted the number of school-age students in urban and rural primary schools from 2016-- 2030 and then calculated the required number of teachers while analyzing trends in educational resource demand. Similarly, Hong et al. [27] calculated the surplus and required preschool teachers in urban and rural areas on the basis of the allocation indicators of kindergarten work regulations.

#### Demographic and population change considerations.

The second category is based on educational and instructional needs, focusing on the specific requirements of teachers in actual classroom practice and directly using these needs as the standard for teacher resource allocation. This approach ensures that teacher resources are allocated scientifically and efficiently to meet the real demands of the education system. For example, Wang et al. [28] provide a significant direction for policy creators, who need insights to create better preschool teacher development programs together with resource allocation strategies for Shanghai from 2023 until 2050. Wang et al. [29] predicted the structure of the rural teaching workforce by assessing disparities in teaching workload requirements across different subjects.

#### Multifactor approaches to teacher allocation.

A third type of method is based on composite indicators that include many aspects of education, such as geographical coverage, pupil‒teacher ratios and class sizes. The varying geographical scope leads to differences in the demand for the allocation of educational resources across diverse geographical regions. The number of students per teacher serves as a key statistic for evaluating the adequacy or insufficiency of teaching resources. For example, Yang et al. [10] used student-teacher ratio data to forecast the future need for teachers and reported that the ratio has been decreasing in private schools. The teacher distribution shows sharp contrasts between urban and rural education. In western China, the teacher shortage in the rural education system stems from difficulties such as geographic isolation, poor facilities, and low pay. Teachers are lured to the more comfortable, urban educational sector. To address this problem, incentives have been established to encourage teachers to move to the West. Moreover, local training initiatives have been reemphasized. Finally, the assignment of teachers has become more flexible. Together, these policies seek to bridge the profound educational chasm between the unequal halves of the warm [30].

### Challenges and gaps in existing teacher allocation models

Despite improved teacher allocation methods, challenges remain, especially in dynamic contexts affected by demographic trends and policy changes. Most traditional models assume stable student populations and a fixed teacher supply. In western China, however, student enrollment numbers can shift quickly due to migration, booming economies and changes in government policy. Research that suggests allocation frameworks that are more responsive and data driven will need to be in place to provide “real-time” adjustments to the recruitment and distribution of “leverage” for teachers [31].

Without reliable data on teachers and their allocation, we cannot devise effective plans. However, in rural areas, data gathering remains patchy owing to administrative and infrastructural constraints. There are no accurate demographic or workforce data, which makes predictive models less effective [32]. Proper data collection mechanisms and automated data processing systems can increase the accuracy of forecasts.

The rural retention of teachers faces a number of sociocultural challenges, such as language diversity and lifestyle differences, that can affect the relationships between teachers and students. However, these are not the only issues. Several researchers have identified insufficient access to professional development as a resource in rural settings, especially for minority teachers in communities that have few educational professionals who look or sound like the students they teach [15,17,33]. Inadequate access to professional development might tend to make some teachers, especially those who are new to the profession or the community, feel somewhat isolated and without a clear sense of direction, which might lead them to consider other opportunities.

Overall, the reallocation of educational resources is thus very much attached to the sliding school-age population. Current research adopts mathematical modeling approaches to estimate the demand for educational resources, especially in the context of population changes leading to fluctuations in educational demand, where there is a recognition of high stability and predictive performance. However, a majority of existing research studies perform static analyses of macrolevel planning and forecasting of educational resources. They fail to account for long-term population dynamics in detail and are specific to regions and different stages of school.

The shortage of the school-age population and increasing urbanization in western China have resulted in significant and complex changes in educational resource allocation to the region, which makes the problem more serious. Conventional static analysis techniques do not adequately reflect the dynamic transformation of demand structures and changes among population trends. Additionally, western China faces not only relatively lagging economic development but also significant diversity in population structure, including varied ethnic groups and distinct cultural backgrounds. The interplay of unique social, economic, and geographical factors further complicates the rational allocation of educational resources.

Therefore, moving beyond traditional static models and adopting a more flexible, comprehensive, and dynamic analytical approach are essential. This requires considering the combined effects of regional differences, urban–rural disparities, and population changes on educational resource allocation while developing more targeted policy measures through region-specific forecasting methods. In line with this objective, this study integrates the Leslie model with the age-specific progression rate method, utilizing data from the seventh national census and relevant statistical yearbooks from various regions as population benchmark data. It systematically analyzes and predicts the demand structure for rural preschool, primary, and junior high school teachers across 12 regions in western China, ultimately proposing corresponding adaptation strategies and policy recommendations to address gaps in research on educational resource allocation in western China.

## Research design

### Research subjects

This study focuses on predicting the demand structure for rural teachers in western China on the basis of trends in population change. The Leslie model is used to forecast the preschool, primary, and junior high school-age populations across 12 western regions (including Chongqing, Sichuan, Yunnan, Guizhou, Tibet, Shaanxi, Gansu, Ningxia, Qinghai, Xinjiang, Inner Mongolia, and Guangxi) from 2024-- 2040, accurately estimating the future trends in the school-age population for each region. These 12 provinces were selected on the basis of their designation under China’s Western Development Strategy. They share common rural education challenges—such as geographic remoteness, population outmigration, and development gaps—while also offering internal diversity for meaningful comparative analysis. The number of students at each educational stage is calculated as benchmark data via the age-specific progression rate method. On the basis of these calculations, the required scale and structural characteristics of the preschool, primary, and junior high school teaching workforce in each region are determined, systematically revealing the demand structure and future development trends of rural teacher resources in western China.

### Data sources

The data for this study are derived primarily from the Seventh National Population Census, as well as birth rates, mortality rates, and the natural population growth rates recorded in the statistical yearbooks of various provinces from 2000--2023. To ensure consistency between the research data and the total population figures for each region as of the end of 2023, as announced by the National Bureau of Statistics, and to account for the time difference between the census reference point and the statistical data, a time series regression model was used to fit the population data from 2010--2022 and extrapolate it to 2023. This adjustment ensures alignment with the official total population data released by the National Bureau of Statistics and serves as the benchmark population dataset for this study. The data used for analyzing the current status of rural teachers are sourced from the 2022 education statistics published by the Ministry of Education of the People’s Republic of China, which include specific regional data.

### Research methods and model selection

#### Lesie model.

The Malthusian model and the logistic model in population forecasting primarily predict the total population on the basis of the natural population growth rate but do not account for changes in population structure [11]. To address this limitation, Patrick H Leslie [34] proposed the Leslie model, which not only captures the overall population growth trend but also effectively predicts structural changes across different age groups. Accordingly, this study employs the Leslie model to forecast the future population structure of the western region via population data from 2000--2023.

Since childbearing is exclusive to women, this study considers only the female population. According to the definition of the United Nations Population Fund (UNFPA), the childbearing age of women is generally considered to be between 15 and 49 years [35]. Similarly, on the basis of the research of scholar Bongaarts (2010), this age range is confirmed as the standard reproductive age group for women.

Therefore, this study categorizes women aged 49 years and younger into one-year age groups and divides the analysis period into one-year intervals. The number of female populations in t=1,2,.… the i-th group in $n_{i}(t)$time period t is i=1,2,3...,k, where k = 50. The distribution vector of the female age group in time period t is shown in formula (1).


$${\left[n_{\text{1}}(t),n_{\text{2}}(t),...,n_{k}(t)\right]}^{T}$$ (1)

Assume that the fertility rate of the *i*th age group is $b_{i}$ and that the proportion of female newborns in the total number of new-borns is $f_{i}$. The mortality rate of the *i*th age group is $d_{i}$, and the survival rate is $s_{i}=\text{1}-d_{i}$. In period (t + 1), the number of females in the first age group is the sum of the number of female newborns born to women of childbearing age in the previous period (i.e., period t). Similarly, the number of females in the (t + 1)-th age group is equal to the number of females in the i-th age group who survived in period t. Therefore, we derive formulas (2) and (3).


$$n_{\text{1}}(t+\text{1})=\sum_{i=\text{1}}^{m}f_{i}(t)b_{i}(t)n_{i}(t)$$ (2)

$$n_{i+\text{1}}(t+\text{1})=s_{i}(t)n_{i}(t),i=\text{1},\text{2},...,k-\text{1}$$ (3)

Additionally, formula (4) builds on these equations to refine the estimation of population dynamics across multiple time periods.


$$L(t)=[\begin{array}{ccccc} f(t)b_{\text{1}}(t) & f(t)b_{\text{2}}(t) & ... & f(t)b_{k-\text{1}}(t) & f(t)b_{k}(t)\\ s_{\text{1}}(t) & \text{0} & ... & ... & \text{0}\\ \text{0} & s_{\text{2}}(t) & \text{0} & ... & \text{0}\\ ... & ... & ... & ... & ...\\ \text{0} & ... & ... & s_{k-\text{1}}(t) & ... \end{array}]$$ (4)

Therefore, the total female population in period *t* is denoted as n(t)=L(t−1)n(t−1) in formula (4). Using the Leslie model, we calculate the female population vector in the initial period, which allows us to determine the female population distribution vector for the next period. By incorporating the sex ratio, we can estimate the male population, thereby obtaining the total population for the subsequent period.

#### Age-specific progression rate method.

The grade promotion ratio method predicts the future number of students enrolled in school on the basis of the number of students in lower grades and the promotion rate [36]. The specific calculation formulas are as follows.


$${PSN}_{y}={PSN}_{y-\text{1}}-{PSN}_{y-\text{1}}^{\text{6}}+{EGS}_{y}^{eligible}$$ (5)

$${SSN}_{y}={SSN}_{y-\text{1}}-{PSN}_{y-\text{1}}^{\text{3}}+{aPSN}_{y-\text{1}}^{\text{6}}$$ (6)

Formula (5) represents the number of primary school students in year y, where ${\text{PSN}}_{\text{y}-\text{1}}$ represents the number of primary school students in the previous year, ${\text{PSN}}_{\text{y}-\text{1}}^{\text{6}}$ represents the number of sixth-grade students in the previous year, and ${\text{EGS}}_{\text{y}}^{\text{eligible}}$ represents ${\text{PSN}}_{\text{y}}$ the number of primary school-age children (6 years old) enrolling in primary school that year.

Formula (6) represents ${\text{SSN}}_{\text{y}}$, which denotes the number of junior high school students in year *y*. ${\text{SSN}}_{\text{y}-\text{1}}$ represents the number of junior high school students in the previous year, ${\text{PSN}}_{\text{y}-\text{1}}^{\text{3}}$ represents the number of students in the third grade of junior high school in the previous year, and a represents the admission rate from elementary school to junior high school. Additionally, ${\text{PSN}}_{\text{y}-\text{1}}^{\text{6}}$ represents the number of sixth-grade primary school students in the previous year.

The future population age structure is projected on the basis of the Leslie model combined with the grade progression ratio method. The population data for ages 3–6 (preschool), 6–12 (primary school), and 13–15 (junior high school) are extracted as input parameters for the age-specific progression rate method, which is then used to estimate the student population at each educational stage, leading to Formula (7).


$$S_{y}=S_{y-\text{1}}\times P+I$$ (7)

In Formula (7), ${\text{S}}_{\text{y}}$ represents the number of students in a specific grade in year y, ${\text{S}}_{\text{y}-\text{1}}$ denotes the number of students in the same grade in the previous year, P is the grade progression rate (advancement rate), and I represents the newly enrolled students, typically the number of 6-year-old children, as provided by the Leslie model.

### Model assumptions and parameter settings

#### Model assumptions.

This study utilizes rural data from various regions in western China, as reported in the Seventh National Census, as the population benchmark data. Considering that the core of population forecasting is to estimate demographic changes over a specific period on the basis of the fertility rate, mortality rate, and migration rate, the following model assumption is proposed. First, it is assumed that fertility behavior occurs only among women aged 15--49. Second, it is assumed that no major disasters will occur during the forecast period and that medical standards will remain stable, meaning that the age-specific mortality rate will not change significantly. Third, the domestic population is considered a closed system, and the effects of domestic and foreign migration are not accounted for. Finally, in accordance with the Guidelines for Learning and Development of Children aged 3--6 years, the preschool education age group was defined as 3--6 years. Similarly, according to the Compulsory Education Law of the People’s Republic of China, the school-age population for compulsory education is defined as those aged 6--15 years, with those aged 6--12 years corresponding to primary school and those aged 13--15 years corresponding to junior high school.

#### Birth rate.

This study applies time series analysis to estimate the birth rate from 2024-- 2040 on the basis of birth rate data from provinces (municipalities, autonomous regions) in western China collected between 2000 and 2023. By collecting and analyzing historical data, identifying the long-term trend of the birth rate, using regression analysis for prediction, and considering the impact of potential policy changes and socioeconomic factors on the birth rate, the prediction results are ultimately used as input parameters for the population prediction model.

#### Population mortality rate.

This study uses time series analysis to estimate the mortality rate from 2024-- 2040 on the basis of the mortality data of each province (municipality, autonomous region) from 2000--2023. By analyzing historical data, evaluating the mortality rate trend, using regression analysis for prediction, and considering the impact of factors such as aging, medical progress, and public health policies, the mortality rate prediction results can be used as input parameters and combined with birth rate data to predict future population changes.

#### Number of first-grade primary school students.

According to the Compulsory Education Law of the People’s Republic of China, “For children who are six years old or older, their parents or other legal guardians shall send them to school to receive and complete compulsory education.” On the basis of this provision, this study assumes that the number of six-year-old school-age children each year is equivalent to the number of first-grade primary school students in that year.

#### Dropout rate.

According to the Compulsory Education Law of the People’s Republic of China, “Compulsory education is an education that all school-age children and teenagers must receive, which is uniformly implemented by the state.” Therefore, this study assumes that all school-age children will complete the full nine years of compulsory education, and the dropout rate is considered to be zero. While we assume zero dropout in line with national policy, localized dropout in rural areas may occur. This assumption represents an upper-bound scenario; actual teacher demand may be slightly lower.

#### Admission rate.

According to the Statistical Bulletin of Education Development, China’s average primary school net enrollment rate from 2013--2022 was 99.91%. Using FORECAST. The ETS function—which applies exponential smoothing based on historical values to predict future values—in this study estimates the primary school enrollment rate from 2024-- 2040. The projection indicates that the net enrollment rate will reach 100% by 2024; therefore, this study assumes that the primary and secondary school enrollment rates are 100%. Similarly, according to the Statistical Bulletin of Education Development, China’s average junior high school net enrollment rate from 2013--2022 was 101.6%. Therefore, the junior high school enrollment rate in this study is also assumed to be 100%.

#### Urbanization trend estimation.

The forecast of rural student populations accounts for urbanization by estimating the concentration rate of students in rural schools. We applied a linear extrapolation model, following Yuyou and Xiaohua (2017), to calculate the average annual decline in rural student proportions from 2000--2023 for each education level. This approach reflects the continuing trend of educational centralization and rural-to-urban migration, allowing us to project the rural share of total students from 2024-- 2040. These estimated rural concentration rates were then applied to the projected total student numbers to determine rural student populations across preschool, primary, and junior high levels.

## Results

### Forecast results for the rural school-age population in 12 regions in western China from 2024-- 2040

**Fig 1:**
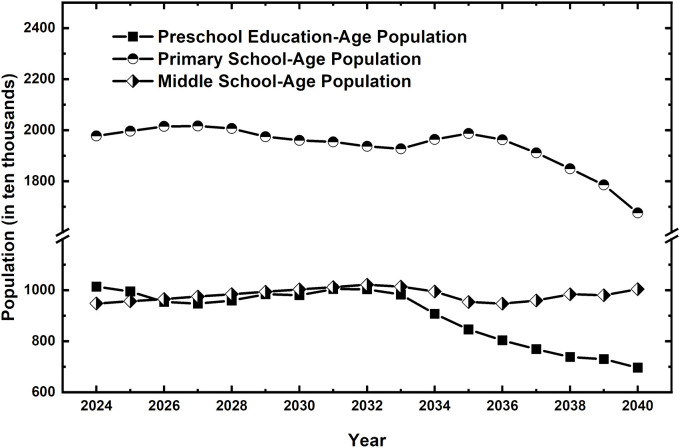
Projected school-age population (aged 3–15) in 12 western provinces of China, 2024–2040.

This study defines the preschool-aged population as 3--6 years old and the compulsory education-aged population as 6--15 years old, corresponding to grades 1--9 of the compulsory education stage. Considering cross-age factors within the school year system, the number of students in each grade is calculated as the two-year average of the corresponding age group. For example, children in the senior preschool class are 5 or 6 years old, so the number of children in this class is calculated as the average of the 5-year-old and 6-year-old populations. Similarly, first-grade primary school students are 6 or 7 years old, so the number of first-grade students is the average of the 6-year-old and 7-year-old populations. To simplify the chart, the number of children in the senior preschool class is represented as 5 years old, and the number of first-grade students is represented as 6 years old. However, the actual calculations are based on the corresponding two-year average, and the same approach is applied to other grades (Fig 1).

The number of birth years for the school-age population aged 3--15 between 2024 and 2040 ranges from 2008--2037. On the basis of Table 1, Fig 1 illustrates the forecasted school-age population at each educational stage between 2024 and 2040 via the population forecast model. For example, first-grade primary school students in 2028 correspond to the birth years of 2021--2022, with a forecasted population of 3.57 million. By collecting statistical yearbook data from various provinces (municipalities), the actual recorded number of births is 3.217 million, which is closely aligned with the forecasted newborn population (Table 1).

**Table 1:** Prediction of the total population of preschool, primary and junior high school-aged children in 12 regions in western China from 2024-- 2040 (Unit: 10,000 people).

Year Age	3	4	5	Total preschool education-age population	6	7	8	9	10	11	Total primary school-age population	12	13	14	Total middle school-age population
2024	329.4	343.5	340.4	1013.3	337.4	334.3	331.2	328.1	325.0	321.6	1977.6	318.9	315.8	312.8	947.5
2025	321.7	329.4	343.5	994.6	340.4	337.4	334.3	331.2	328.1	325.0	1996.4	321.6	318.9	315.8	956.3
2026	302.4	321.7	329.4	953.5	343.5	340.4	337.4	334.3	331.2	328.1	2014.9	325.0	321.6	318.9	965.5
2027	322.8	302.4	321.7	946.9	329.4	343.5	340.4	337.4	334.3	331.2	2016.2	328.1	325.0	321.6	974.7
2028	334.3	322.8	302.4	959.5	321.7	329.4	343.5	340.4	337.4	334.3	2006.7	331.2	328.1	325.0	984.3
2029	326.5	334.3	322.8	983.6	302.4	321.7	329.4	343.5	340.4	337.4	1974.8	334.3	331.2	328.1	993.6
2030	319.3	326.5	334.3	980.1	322.8	302.4	321.7	329.4	343.5	340.4	1960.2	337.4	334.3	331.2	1002.9
2031	358.4	319.3	326.5	1004.2	334.3	322.8	302.4	321.7	329.4	343.5	1954.1	340.4	337.4	334.3	1012.1
2032	325.7	358.4	319.3	1003.4	326.5	334.3	322.8	302.4	321.7	329.4	1937.1	343.5	340.4	337.4	1021.3
2033	298.4	325.7	358.4	982.5	319.3	326.5	334.3	322.8	302.4	321.7	1927.0	329.4	343.5	340.4	1013.3
2034	283.1	298.4	325.7	907.2	358.4	319.3	326.5	334.3	322.8	302.4	1963.7	321.7	329.4	343.5	994.6
2035	264.2	283.1	298.4	845.7	325.7	358.4	319.3	326.5	334.3	322.8	1987.4	302.4	321.7	329.4	953.5
2036	255.9	264.2	283.1	803.2	298.4	325.7	358.4	319.3	326.5	334.3	1962.6	322.8	302.4	321.7	946.9
2037	248.6	255.9	264.2	768.7	283.1	298.4	325.7	358.4	319.3	326.5	1911.4	334.3	322.8	302.4	959.5
2038	233.5	248.6	255.9	738.2	264.2	283.1	298.4	325.7	358.4	319.3	1849.1	326.5	334.3	322.8	983.6
2039	247.6	233.5	248.6	729.7	255.9	264.2	283.1	298.4	325.7	358.4	1785.7	319.3	326.5	334.3	980.1
2040	215.3	247.6	233.5	696.4	248.6	255.9	264.2	283.1	298.4	325.7	1675.9	358.4	319.3	326.5	1004.2

The primary school-age population is expected to gradually decline from 19.776 million in 2024 to 16.759 million in 2040, representing a 15.2% decrease. Although demand for primary education remains relatively stable in the short term, the long-term downward trend is evident and should not be overlooked.

The junior high school-age population, however, is projected to increase from 9.475 million in 2024 to 10.042 million in 2040, showing a slight growth trend. This increase is related to the larger kindergarten and primary school-age populations in previous years, indicating that the demand for education in the later stages of compulsory education will persist. The current junior high school student population is directly linked to the past accumulation of kindergarten and primary school enrollments. While this increase highlights the continuity of school-age population dynamics, the long-term decline in preschool and primary school populations suggests that this growth trend may slow over time. Therefore, the development of a rural teaching workforce must fully consider the dynamic relationship between population mobility and urbanization trends to ensure an adequate and well-distributed supply of teachers in the future.

### Forecast results of the number of students in rural areas of 12 regions in western China from 2024-- 2040

The urban‒rural composition of school enrollment is determined by the total number of students enrolled and the education urbanization rate. To predict the scale of rural school enrollment in 12 regions in western China, it is necessary to systematically estimate the education concentration rate at each stage on the basis of trends in the school-age population and the progress of education urbanization. The term ‘education concentration/urbanization rate’ refers to the proportion of students enrolled in urban schools relative to the total student population in a province. This rate captures the extent of educational urbanization, which has implications for resource planning and teacher allocation. Following the prediction method of Yuyou and Xiaohua [37], a linear decline model is used to calculate the average annual decline rate by integrating historical data on the rural concentration of primary and junior high school students from 2000--2023. The specific calculation and prediction results are as follows (Table 2).

**Table 2:** Concentration of preschool education, primary school education and junior high school education in rural China from 2024-- 2040.

Year	Preschool rural concentration (%)	Primary school rural concentration (%)	Middle school rural concentration (%)
2024	17.68	19.04	9.21
2025	16.79	18.67	8.96
2026	15.94	18.3	8.71
2027	15.12	17.93	8.46
2028	14.34	17.56	8.21
2029	13.59	17.19	7.96
2030	12.88	16.82	7.71
2031	12.2	16.45	7.46
2032	11.55	16.08	7.21
2033	10.94	15.71	6.96
2034	10.35	15.34	6.71
2035	10.13	14.97	6.46
2036	9.85	14.6	6.21
2037	9.43	14.23	5.96
2038	8.78	13.86	5.71
2039	8.46	13.49	5.46
2040	8.15	13.12	5.21

In view of the continuous decline in the natural population growth rate and the steady advancement of urbanization, the number of students in rural preschool education, primary school, and junior high school has significantly decreased. This decline is particularly evident in preschool education, where the rural concentration decreased from 17.68% in 2024 to 8.15% in 2040, representing a decrease of more than 50%. This sharp decline reflects the direct impact of the rapid reduction in student numbers on the demand for rural preschool education resources. Similarly, the rural concentration of primary schools has fallen from 19.04% in 2024 to 13.12% in 2040, a decrease of approximately 31%. Moreover, the rural concentration of junior high schools has decreased from 9.21% to 5.21%, indicating a 43% decrease. These trends indicate that the concentration of basic education resources in urban areas has become an irreversible process, posing a structural challenge to the sustainable development of rural education.

**Fig 2:**
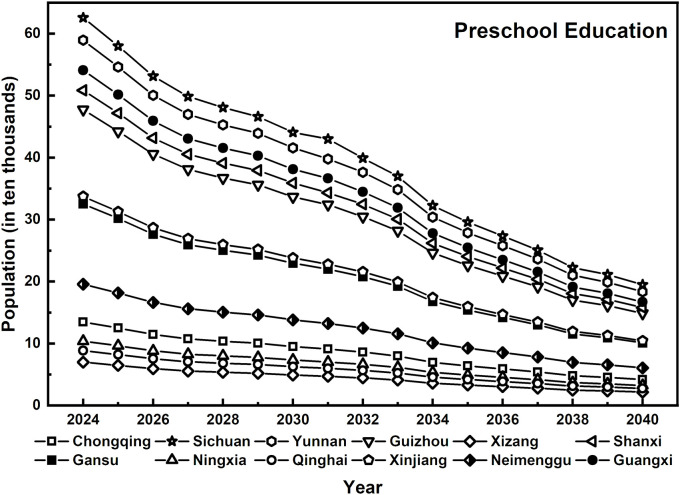
Forecasted rural preschool enrollment in western China, 2024–2040.

On the basis of the forecast of rural education concentration trends in preschool, primary, and junior high schools, combined with projections of student enrollment in preschool and compulsory education, the forecasted trend of the rural student population in western China is obtained. The number of students in preschool education has declined significantly (as shown in Fig 2), with an overall decrease of more than 50%. For example, in Sichuan, enrollment has decreased from 625,600 in 2024–194,700 in 2040, a decrease of 68.9%. Similarly, Yunnan’s enrollment has declined from 589,500–183,400, reflecting a nearly 69% decrease. This trend underscores the profound impact of declining birth rates on the rural preschool-aged population and highlights the intensifying migration of rural populations to cities. As a result, demand for rural preschool education has shrunk rapidly, and the need for preschool teachers has continued to decline.

**Fig 3:**
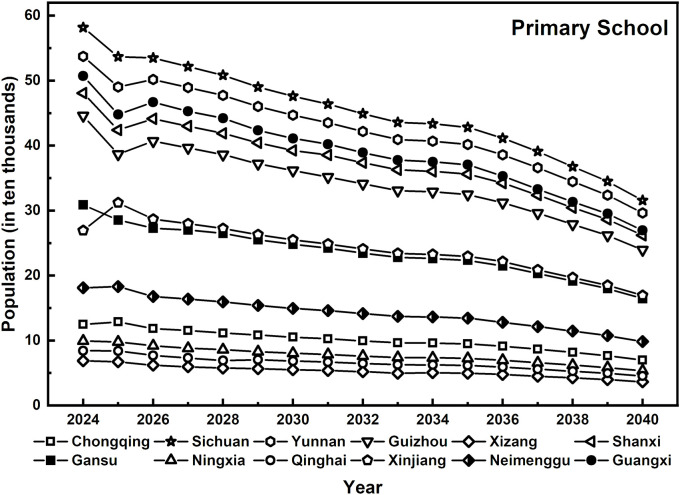
Projected rural primary school enrollment across 12 western provinces, 2024–2040.

Although the decline in the number of rural primary school students is less severe than it is in the preschool education stage, the overall trend remains notable. The increasing concentration of educational resources in urban areas has exacerbated the challenges faced by rural basic education due to resource depletion (as shown in Fig 3). For example, the number of primary school students in Chongqing has decreased from 134,800 in 2024–42,000 in 2040, a decrease of 68.8%, indicating that rural basic education is experiencing significant student attrition and an increasing agglomeration of educational resources in urban centers. Similarly, in Tibet, the number of rural primary school students has declined from 69,900 in 2024–21,600 in 2040. Although this represents a 69.1% decrease, its already small population base, combined with geographical and economic constraints, poses unique challenges for teacher allocation. Overall, these changes in rural primary school enrollment reflect the tendency of students to migrate toward urban areas during the urbanization process. For remote and sparsely populated regions, the challenge of allocating basic education resources, particularly teachers, becomes even more severe.

**Fig 4:**
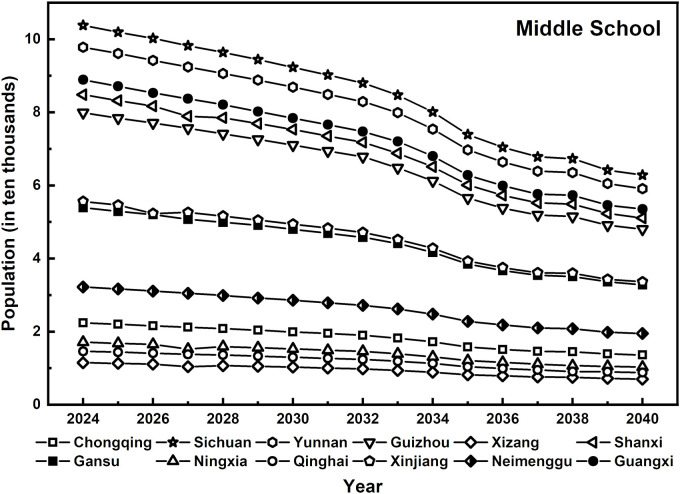
Forecast of rural junior high school enrollment, 2024–2040.

Although the overall decline in the number of rural junior high school students is comparable to that of preschool and primary school students, the inelastic demand has resulted in different pressures on educational resources (as shown in Fig 4). For example, the number of rural junior high school students in Gansu is projected to decrease from 325,300 in 2024–101,000 in 2040, a decrease of 68.9%. Similarly, in Qinghai, the number decreases from 88,500–27,400, reflecting a nearly 69% decrease.

Despite the similar rate of decline, as the final stage of compulsory education, junior high school demand remains relatively stable due to its rigid characteristics. This stability ensures a more consistent teacher supply at this stage, in contrast to the rapid reductions in teacher demand seen in preschool and primary education due to declining student numbers. However, with the gradual decline in student numbers at earlier educational stages, junior high school education, while still maintaining relative stability in the short term, will inevitably be affected by the long-term decline in the school-age population. As a result, pressure on teacher allocation and resource distribution will become increasingly evident in the future.

Overall, the trends in the number of rural students in preschool education, primary school, and junior high school across 12 regions in western China from 2024-- 2040 exhibit a gradient from a significant decline to a relatively stable trend. This pattern highlights the impact of population structure adjustments and the restructuring of rural education demand driven by urbanization, necessitating a reassessment of rural teacher allocation strategies.

The sharp decline in preschool enrollment underscores the need for a teacher allocation strategy that balances centralization with flexibility. At the primary school level, the supply of educational resources must prioritize both disciplinary balance and teacher quality improvement, particularly in addressing the loss and replacement of foundational teachers caused by disparities in urban and rural educational resources [38]. Although the decline in demand for junior high school education is relatively slow, long-term stability and the sustained service capacity of the teaching workforce remain critical considerations [39]. Therefore, the development of the rural teacher workforce must align with the evolving student population trends at each educational stage, implementing refined and differentiated regulation strategies. This approach will enable rural education systems to navigate the complex challenges arising from shifting educational resource demands, ultimately ensuring a dynamic balance between education supply and demand.

The projected decline in rural student numbers across all levels—most notably in preschool education—clearly signals the need to recalibrate teacher allocation strategies. These student trends serve as the foundation for estimating future teacher demand. Accordingly, the following section builds upon these findings by forecasting the demand structure for rural teachers across different education stages in the 12 western provinces.

### Projected demand structure for rural teachers (2024–2040)

According to the notice on unifying the staffing standards for primary and secondary schools in urban and rural areas, which were jointly issued by the Central Organization Department, the Ministry of Education, and the Ministry of Finance in 2014, the teacher‒student ratio standards for primary and junior high schools are 1:19 and 1:13.5, respectively. Additionally, according to the Provisional Standards for Staffing Kindergarten Teachers, Kindergarten teaching and support staff include full-time teachers, childcare workers, administrative staff, and teaching assistants. Among these roles, full-time teachers are the primary focus because of their essential role in the development of preschool education. Therefore, this study focuses on forecasting the demand for full-time teachers and refers to the teacher‒student ratio parameters established by Sha et al. [40]. With a teacher‒student ratio of 1:12, the specific teacher demand calculations for each stage are obtained.

Owing to factors such as the declining school-age population, the continuous decrease in birth rates, and the acceleration of urbanization, the number of rural preschool teachers in western China has sharply decreased (see Table 3). For example, the demand for rural preschool teachers in Sichuan is projected to decrease from 625,600 in 2024–194,700 in 2040, whereas in Qinghai, it is expected to decline from 88,500–27,400, reflecting reductions of 68.9% and 69.0%, respectively. This significant decline is directly linked to the sharp drop in the school-age population, particularly in densely populated provinces such as Sichuan and Guizhou, where decreasing birth rates have led to a substantial decline in demand for preschool education. Similarly, provinces with smaller populations, such as Qinghai, are experiencing a decrease in rural preschool teacher demand, driven by both declining birth rates and population loss. Against the backdrop of accelerated urbanization, the growing imbalance in preschool education resources has become increasingly evident. Therefore, adjusting teacher allocation strategies to optimize resource utilization and ensure the continued stability of education quality is urgently needed.

**Table 3:** Forecast of rural preschool teacher numbers in 12 regions in western China from 2024-- 2040 (Unit: 10,000 people).

Year Region	Chongqing	Sichuan	Yunnan	Guizhou	Xizang	Shaanxi	Gansu	Ningxia	Qinghai	Xinjiang	Neimenggu	Guangxi
2024	13.48	62.56	58.95	47.73	6.99	50.85	32.53	10.38	8.85	33.73	19.55	54.09
2025	12.50	57.99	54.61	44.23	6.47	47.17	30.2 0	9.63	8.21	31.29	18.14	50.16
2026	11.45	53.15	50.06	40.57	5.93	43.17	27.64	8.82	7.53	28.68	16.63	45.93
2027	10.75	49.85	46.98	38.10	5.56	40.53	25.94	8.28	7.06	26.93	15.60	43.06
2028	10.36	48.08	45.29	36.70	5.36	39.11	25.06	8.02	6.82	25.96	15.06	41.54
2029	10.05	46.59	43.92	35.61	5.20	37.94	24.28	7.76	6.61	25.18	14.61	40.31
2030	9.51	44.07	41.57	33.65	4.91	35.89	22.96	7.34	6.25	23.81	13.81	38.11
2031	9.10	42.99	39.78	32.40	4.70	34.32	21.98	7.01	5.98	22.79	13.23	36.66
2032	8.61	39.93	37.63	30.45	4.45	32.48	20.79	6.64	5.66	21.56	12.50	34.49
2033	7.98	36.99	34.85	28.20	4.12	30.07	19.23	6.14	5.23	19.92	11.57	31.91
2034	6.95	32.26	30.40	24.63	3.59	26.19	16.77	5.36	4.57	17.39	10.09	27.81
2035	6.39	29.61	27.88	22.60	3.29	24.05	15.39	4.92	4.19	15.94	9.25	25.49
2036	5.90	27.35	25.79	20.89	3.04	22.18	14.20	4.54	3.86	14.68	8.53	23.50
2037	5.41	25.08	23.64	19.16	2.79	20.35	13.01	4.16	3.53	13.45	7.82	21.56
2038	4.80	22.25	21.2	16.99	2.48	18.06	11.54	3.69	3.14	11.93	6.94	19.12
2039	4.55	21.14	19.89	16.12	2.35	17.11	10.93	3.49	2.97	11.29	6.56	18.09
2040	4.21	19.47	18.34	14.86	2.16	15.79	10.10	3.23	2.74	10.43	6.06	16.69

The demand for rural primary school teachers has steadily declined (as shown in Table 4). Although primary education remains the foundation of rural basic education, the declining school-age population and increasing urbanization have also contributed to a gradual reduction in teacher demand. For example, the number of rural primary school teachers in Sichuan Province is projected to decrease from 581,700 in 2024–315,700 in 2040, whereas in Yunnan Province, the number decreases from 537,300–296,400, reflecting decreases of 45.9% and 44.9%, respectively. These figures highlight the continued reduction in teacher demand due to the shrinking school-age population. However, considering the fundamental role of primary education and the need for equitable educational resources, the decline in teacher demand remains relatively steady. Effectively redistributing primary school teachers, optimizing educational resource allocation, and preventing both teacher surpluses and shortages in certain areas are critical to ensuring educational quality and fairness.

**Table 4:** Forecast of rural primary school teacher numbers in 12 regions in western China from 2024-- 2040 (Unit: 10,000 people).

Year Region	Chongqing	Sichuan	Yunnan	Guizhou	Xizang	Shaanxi	Gansu	Ningxia	Qinghai	Xinjiang	Neimenggu	Guangxi
2024	12.47	58.17	53.73	44.6	6.88	48.08	30.9 0	9.94	8.42	26.92	18.12	50.7 0
2025	12.86	53.66	49.03	38.66	6.72	42.4 1	28.54	9.79	8.39	31.2 0	18.29	44.8 0
2026	11.83	53.48	50.17	40.67	6.2 2	44.12	27.29	9.2 0	7.69	28.68	16.76	46.67
2027	11.54	52.15	48.93	39.64	5.95	43.21	27.01	8.81	7.31	27.98	16.36	45.27
2028	11.14	50.83	47.73	38.61	5.75	41.89	26.49	8.59	6.91	27.25	15.94	44.21
2029	10.83	49.01	46.02	37.2	5.67	40.43	25.52	8.28	7.04	26.3 2	15.38	42.35
2030	10.52	47.6 0	44.69	36.16	5.5 0	39.26	24.82	8.04	6.84	25.5 1	14.93	41.11
2031	10.26	46.38	43.52	35.17	5.37	38.55	24.22	7.85	6.68	24.86	14.6	40.22
2032	9.93	44.91	42.18	34.11	5.2 0	37.37	23.45	7.6 0	6.47	24.10	14.13	38.92
2033	9.64	43.58	40.93	33.07	4.96	36.28	22.8 0	7.39	6.28	23.41	13.7 0	37.77
2034	9.6 1	43.35	40.68	32.89	5.03	36.04	22.62	7.34	6.25	23.26	13.62	37.5 0
2035	9.48	42.82	40.18	32.47	4.96	35.62	22.35	7.25	6.16	22.95	13.44	37.06
2036	9.12	41.13	38.58	31.22	4.77	34.23	21.47	6.96	5.90	22.16	12.79	35.3 0
2037	8.66	39.10	36.60	29.61	4.51	32.39	20.30	6.58	5.59	20.86	12.13	33.29
2038	8.16	36.75	34.47	27.88	4.25	30.45	19.14	6.21	5.27	19.68	11.44	31.34
2039	7.66	34.5 0	32.38	26.18	3.99	28.63	17.98	5.83	4.96	18.49	10.75	29.52
2040	6.98	31.57	29.64	23.95	3.64	26.2 0	16.44	5.34	4.53	16.92	9.83	26.96

The demand for junior high school teachers also shows a yearly decline (as shown in Table 5). Although the reduction is slower than that in preschool and primary education, it still reflects the impact of a shrinking school-age population and the outmigration of rural students to educational resources. For example, in 2024, the demand for rural junior high school teachers was 103,800 in Sichuan, 97,800 in Yunnan, and 22,400 in Chongqing. However, owing to the continued decline in the school-age population and the outflow of rural residents, projections estimate that by 2040, teacher demand will decrease by 62,800 in Sichuan, 59,100 in Yunnan, and 13,600 in Chongqing, reflecting reductions of 39.7%, 39.4%, and 39.3%, respectively. This trend illustrates the sharp decline in teacher demand caused by the shrinking rural school-age population. In populous provinces such as Sichuan and Yunnan, the continuous decline in birth rates and the ongoing urbanization process are the primary drivers of the reduced demand for junior high school teachers.

**Table 5:** Forecast of rural junior high school teacher numbers in 12 regions in western China from 2024-- 2040 (Unit: 10,000 people).

Year Region	Chongqing	Sichuan	Yunnan	Guizhou	Xizang	Shaanxi	Gansu	Ningxia	Qinghai	Xinjiang	Neimenggu	Guangxi
2024	2.24	10.38	9.78	7.99	1.15	8.48	5.39	1.71	1.46	5.56	3.22	8.89
2025	2.2	10.19	9.61	7.84	1.13	8.32	5.29	1.68	1.44	5.46	3.17	8.71
2026	2.16	10.02	9.42	7.71	1.11	8.17	5.2 0	1.65	1.41	5.23	3.11	8.53
2027	2.12	9.82	9.24	7.56	1.04	7.89	5.07	1.52	1.38	5.26	3.05	8.37
2028	2.08	9.64	9.06	7.41	1.07	7.85	4.99	1.59	1.36	5.16	2.99	8.21
2029	2.04	9.44	8.88	7.26	1.05	7.69	4.91	1.56	1.33	5.05	2.92	8.02
2030	1.99	9.23	8.69	7.1 2	1.03	7.53	4.8	1.53	1.3 0	4.94	2.86	7.84
2031	1.95	9.02	8.49	6.94	1.00	7.35	4.69	1.49	1.27	4.83	2.79	7.66
2032	1.9 0	8.8 0	8.29	6.78	0.98	7.18	4.58	1.46	1.24	4.71	2.72	7.47
2033	1.82	8.47	7.99	6.48	0.94	6.88	4.41	1.39	1.19	4.52	2.62	7.20
2034	1.72	8.01	7.54	6.12	0.89	6.51	4.17	1.31	1.13	4.28	2.48	6.80
2035	1.58	7.39	6.97	5.65	0.82	6.01	3.87	1.21	1.04	3.93	2.28	6.28
2036	1.51	7.04	6.64	5.38	0.79	5.73	3.67	1.16	0.99	3.75	2.18	5.99
2037	1.46	6.78	6.39	5.19	0.76	5.52	3.54	1.11	0.95	3.61	2.10	5.76
2038	1.45	6.73	6.35	5.15	0.75	5.49	3.51	1.07	0.91	3.60	2.08	5.73
2039	1.39	6.42	6.05	4.91	0.72	5.23	3.36	1.05	0.90	3.43	1.98	5.46
2040	1.36	6.28	5.91	4.8 0	0.7 0	5.11	3.28	1.03	0.88	3.36	1.95	5.35

In rapidly urbanizing areas such as Chongqing, where large numbers of rural residents are migrating to cities, junior high school enrollment is experiencing significant declines. Therefore, the decreasing demand for teachers at the junior secondary education level is not only a reflection of demographic changes but also an inevitable consequence of broader social structural shifts, economic disparities, and widening urban‒rural gaps.

In summary, the changing demand for rural teachers in western China is closely linked to multiple socioeconomic factors, particularly the declining school-age population, continuously decreasing birth rate, and accelerating urbanization process. As the school-age population continues to shrink rapidly, the demand for rural teachers in western China has exhibited a significant downward trend, particularly in preschool education, where the decline has been especially pronounced. While primary and junior high school education have also experienced declines, these reductions have been relatively moderate, reflecting the distinct roles of each educational stage within the system and their varying sensitivity to demographic changes. To ensure effective teacher allocation, more targeted adjustments must be made on the basis of the specific characteristics and actual needs of each educational stage. This study assumes a closed system without modeling interprovincial migration. While data limitations prevent detailed migration analysis, existing national trends show relatively low net migration in most provinces, suggesting minimal impact on our projections.

## Discussion

### Population change trends affect resource allocation in rural areas of western China

On the basis of the trends in the school-age population changes in western China, this paper predicts and analyzes the allocation of educational resources in western China. The ongoing decline in the school-age population is reducing rural student numbers, necessitating structural adjustments in teacher workforce planning. This shift directly influences the distribution of educational resources among preschool, primary, and junior high schools, resulting in an overall decrease in educational demand and thereby causing a mismatch in the distribution of educational resources [11]. The decline of the school-age population may lead to a situation where “schools are difficult to fully fill” in some areas, thereby leading to inefficiency in the allocation of teachers and educational resources [25], especially in some economically backward areas, where the gap between the demand and supply of educational resources is more prominent. The changes in the demand for educational resources caused by the changes in the school-age population require a review of the existing resource allocation mechanism and adjustments based on the new demand background to ensure the rational allocation of educational resources [11,41], thereby improving the quality of education and promoting educational equity and efficient development [38].

### The demand structure for rural teachers shows a continuous downward trend

This study predicts the dynamic changes in the school-age population in western China and conducts an in-depth analysis of the future demand structure for rural teachers across various regions. The findings indicate that, owing to the continuous decline in the school-age population, the demand for teachers in rural preschool education, primary schools, and junior high schools has shown a significant downward trend.

This change directly reflects the impact of the declining school-age population on teacher demand and resource allocation [21,27]. It also highlights variations in teacher demand across different educational stages, with the sharpest decline observed in preschool education, suggesting that this stage faces significant supply pressure. For example, by 2040, the demand for preschool, primary, and junior high school teachers in Sichuan is expected to decline to 194,700, 315,700, and 62,800, respectively.

The reduction in teacher demand poses major challenges for rural areas, particularly with respect to teacher allocation, mobility, and workforce stability [42]. High teacher turnover rates add to instability in certain regions, worsening the imbalance of educational resources and thwarting improvements in the quality of education [43]. Thus, optimizing teacher resource allocation and improving workforce stability under declining demand have become the key issues for the future of rural education.

### Rural teacher structural adaptation path

As the modernization of education in western China continues to accelerate, the demand structure of rural teachers is facing growing challenges. Owing to the prolonged shrinking trend in the school-age population and the imbalance in the allocation of urban and rural education resources, it is necessary to profoundly optimize and adjust the quantity, quality and structure of teachers. It is not only an effective way to solve existing problems but also a scientific and systematic demand structure optimization path for a comprehensive improvement in education quality and equal distribution of educational resources in rural areas.

### Adjusting teacher allocation standards to adapt to population dynamics

Owing to the gradual decline in the rural school-age population in western China, the traditional student‒teacher ratio standard can no longer effectively address the increasingly complex needs of rural education [8]. In response, it is necessary to adjust teacher allocation standards on the basis of population dynamics to ensure the rational distribution of teacher resources and improve teacher quality to better align with evolving educational demands [40]. Particularly in the context of persistently low fertility rates, rural aging and a declining youth population further impact both student enrollment and the age structure of students. Therefore, optimizing the age structure of the teaching workforce and gradually adjusting the allocation standards for full-time teachers at all educational stages is essential. These adjustments should align with the strategic goal of an “education power” nation and reflect trends in population changes, supporting the needs of new urbanization and urban‒rural integration development. This recommendation is consistent with China’s “Special Post Plan” (特岗计划), which recruits and deploys university graduates to teach in rural and remote areas. This nationally coordinated initiative exemplifies how teacher allocation policies can be tailored to regional needs and demographic shifts.

To achieve this goal, it is necessary to establish a comprehensive teacher allocation standard that integrates student-teacher ratios and class-teacher ratios as core principles. Thomas [44] noted that although declining fertility rates may reduce the proportion of young teachers, new teachers can bring fresh perspectives and methods to education while contributing to the professional development of veteran teachers. Therefore, in the face of challenges posed by population mobility and accelerated urbanization, it is necessary to plan systematically and incrementally to recruit high-level and high-quality professional teachers, optimize the age structure of the teaching staff, and better adapt to the long-term development needs of rural education. This will help promote the overall improvement of education quality and further advance educational equity and the balanced allocation of resources.

### Strengthening teachers’ professional development and improving the quality of education and teaching

In light of the gradual decrease in the rural school-age population in western China, the changing population trends and accelerated urbanization process have significantly impacted the allocation of rural education resources, the demand structure for teaching staff, and the assurance of both quantity and quality. Compared with Eastern China, Western China has long faced the challenge of not having enough education resources. Teachers are often stymied by a lack of knowledge of their subject as well as outdated teaching methods as well as a lack of expertise in science and technology education as well as applications in information technology [45]. This makes it difficult to guarantee excellent education.

To address these challenges, the government should establish special education funds to help strengthen weak connections in education in western China. Efforts such as China’s National Teacher Training Program (NTTP) and localized professional development schemes have demonstrated effectiveness in equipping rural educators. Internationally, programs such as “Teach for Nigeria” also illustrate the value of targeted initiatives that improve teacher preparedness and retention in underserved regions. It is essential to prioritize investments in teacher training, infrastructure development and education informatization to ensure that educational resources are accessible to rural areas efficiently. The government must increase the support of rural teachers in western China by focusing on financial and policy incentives, especially in areas of critical importance, such as the use of information technology, interdisciplinary education and modern teaching techniques [18]. The focus of the system should be on enhancing teachers’ professional competence and teaching innovative skills through the use of digital tools, such as online courses, online courses and distance learning, to increase the accessibility of the educational resources available in rural areas and increase the quality of education for all [46].

Additionally, by creating career paths for all employees, providing teachers with opportunities for professional growth, accurately predicting teacher demand gaps, and dynamically allocating resources, the movement and distribution of teacher resources between rural and urban regions and across grade levels can be more effectively coordinated [29]. This, in turn, helps facilitate the rational distribution of educational resources in rural areas, enhancing both educational quality and equity.

### Optimize the teacher training model and enhance the adaptability of education quality

Against the backdrop of increasing population mobility, declining birth rates, and a shrinking school-age population, rural education in western China is facing significant challenges related to teacher attrition. Given the long-term persistence of this issue, digital empowerment should be leveraged to help teachers overcome geographical and resource limitations [47,48], thereby narrowing the educational resource gap between western China and other regions.

Teacher education in rural schools should focus on expanding the scope of education, as well as enhancing teaching methods. Through the use of internet-based platforms to train as well as distance learning tools to expand teachers’ possibilities to better accommodate the changing needs of society in education, teacher training must also be able to accommodate the evolving demands of demographic changes triggered by these changes [33]. Recent digital initiatives—such as China’s “Smart Education of China” platform and the National Public Service Platform for Educational Resources—are already helping bridge urban‒rural education gaps. Likewise, India’s DIKSHA platform provides open-access digital resources and training for teachers, offering a scalable model for rural digital empowerment.

Training should focus on expanding and diversifying the curriculum of fundamental disciplines, with particular focus given to enhancing the ability to teach different disciplines. Enhancing the effectiveness of interactive and differentiated teaching can allow teachers to adapt their teaching methods to meet students’ various needs and expectations for strategies to teach. Leveraging urban resources for education can also improve quality teaching, creating stronger connections between rural and urban educational systems and furthering improvements in the overall quality of education [38].

Junior high school teacher education should focus on expanding teachers’ subject knowledge and creating new teaching strategies across a variety of educational areas. Instructional design and curriculum development must also incorporate digital tools for teaching so that teachers are up-to-date with educational technology while improving their effectiveness when teaching digital classrooms [47]. This method will enable teachers to adapt quickly to an ever-evolving education environment while facing the challenges presented by a decreasing school population efficiently.

## Conclusion

The study is based on Leslie models and the age-specific progression rate method. Various factors, such as changes in population, education concentration and urbanization, are analyzed to determine the need for teachers from rural areas in primary and preschool education schools and junior high schools across 12 areas in western China between 2024 and 2040. In analyzing the demand patterns for teachers in different education levels, this study shows how changing demographics affect teacher resource allocation strategies. The results indicate that the configuration and distribution of teacher resources at each stage urgently require optimization and adjustment. It is essential to develop a diversified teacher training system, enhance resource allocation mechanisms, and implement long-term support strategies to address teacher shortages and the uneven distribution of educational resources in rural western China. Additionally, this study aims to fill the gap in research on the demand structure for rural teachers at different educational stages in western China. Moreover, this study offers a new perspective and theoretical foundation for addressing issues such as the unequal distribution of educational resources and teacher shortages. It also provides scientific support for the refinement of education policies, making it highly relevant for practical implementation. To guide policy, we recommend (1) adjusting rural teacher allocation formulas via demographic forecasts; (2) promoting flexible deployment models, such as multigrade and mobile teaching; and (3) strengthening interagency data coordination for evidence-based workforce planning.

Although 100% enrollment and zero dropout reflect policy goals, real conditions may vary. Future studies should incorporate sensitivity analysis to increase the projection accuracy. However, the population benchmark data used in this study mainly originate from provincial statistical sources, which do not fully capture local variations. As a result, the findings may not fully align with the specific needs of different regions. Future research should prioritize the collection and analysis of localized data to increase the accuracy of teacher demand predictions. Moreover, while this study accounts for multiple factors, including population mobility and urbanization, there are still gaps in teacher allocation standards and the implementation of education policies. Future research should further explore strategies for dynamically adjusting teacher allocation standards in response to regional urbanization trends and population migration patterns, ensuring a more balanced distribution of educational resources and a more precise alignment of teacher supply with demand.

## Supporting information

S1 Data Provincial-level population sample data from the National Population Sample Survey for all provinces of China (2002–2021). (XLSX)

S2 Data Provincial-level crude birth rates, crude death rates, and rates of natural population increase for all provinces of China (2000–2021). (XLSX)
