# Correction: Demand structure prediction and adaptation path of rural teachers in western China under population change

**DOI:** 10.1371/journal.pone.0339499

**Published:** 2025-12-19

**Authors:** Yang Lv, Bingyu Hao, Xuemei Zhang, Min Wu

The second author’s name is spelled incorrectly. The correct name is: Bingyu Hao.
